# *KCNN2* polymorphisms and cardiac tachyarrhythmias

**DOI:** 10.1097/MD.0000000000004312

**Published:** 2016-07-22

**Authors:** Chih-Chieh Yu, Tsai Chia-Ti, Pei-Lung Chen, Cho-Kai Wu, Fu-Chun Chiu, Fu-Tien Chiang, Peng-Sheng Chen, Chi-Ling Chen, Lian-Yu Lin, Jyh-Ming Juang, Li-Ting Ho, Ling-Ping Lai, Wei-Shiung Yang, Jiunn-Lee Lin

**Affiliations:** a Department of Internal Medicine, National Taiwan University Hospital; b Graduate Institute of Clinical Medicine, College of Medicine, National Taiwan University; c Department of Medical Genetics, National Taiwan University Hospital; d Graduate Institute of Medical Genomics and Proteomics, College of Medicine; e Research Center for Developmental Biology and Regenerative Medicine, National Taiwan University, Taipei; f Department of Internal Medicine, National Taiwan University Hospital, Yun-Lin Branch, Yun-Lin, Taiwan; g Krannert Institute of Cardiology and Division of Cardiology, Department of Medicine, Indiana University School of Medicine, Indianapolis, IN, USA; h Graduate Institute of Epidemiology and Preventive Medicine, College of Public Health, National Taiwan University, Taipei, Taiwan.

**Keywords:** association studies, genetics, heart arrest, ion channel, risk prediction, ventricular arrhythmia

## Abstract

Potassium calcium-activated channel subfamily N member 2 (KCNN2) encodes an integral membrane protein that forms small-conductance calcium-activated potassium (SK) channels. Recent studies in animal models show that SK channels are important in atrial and ventricular repolarization and arrhythmogenesis. However, the importance of SK channels in human arrhythmia remains unclear. The purpose of the present study was to test the association between genetic polymorphism of the SK2 channel and the occurrence of cardiac tachyarrhythmias in humans. We enrolled 327 Han Chinese, including 72 with clinically significant ventricular tachyarrhythmias (VTa) who had a history of aborted sudden cardiac death (SCD) or unexplained syncope, 98 with a history of atrial fibrillation (AF), and 144 normal controls. We genotyped 12 representative tag single nucleotide polymorphisms (SNPs) across a 141-kb genetic region containing the KCNN2 gene; these captured the full haplotype information. The rs13184658 and rs10076582 variants of KCNN2 were associated with VTa in both the additive and dominant models (odds ratio [OR] 2.89, 95% confidence interval [CI] = 1.505–5.545, *P* = 0.001; and OR 2.55, 95% CI = 1.428–4.566, *P* = 0.002, respectively). After adjustment for potential risk factors, the association remained significant. The population attributable risks of these 2 variants of VTa were 17.3% and 10.6%, respectively. One variant (rs13184658) showed weak but significant association with AF in a dominant model (OR 1.91, CI = 1.025–3.570], *P* = 0.042). There was a significant association between the KCNN2 variants and clinically significant VTa. These findings suggest an association between KCNN2 and VTa; it also appears that KCNN2 variants may be adjunctive markers for risk stratification in patients susceptible to SCD.

## Abstract 2

Supplemental Digital Content is available in the text

## 1 Introduction

The genetic basis of ventricular tachyarrhythmias (VTa) and sudden cardiac death (SCD) remains unclear. Even when exposed to the same risk factors, such as ischemic or nonischemic cardiomyopathy, inherited channelopathies, or metabolic syndrome, some patients experience lethal arrhythmias while others have minimal symptoms.^[1–4]^ These findings suggest that unknown disease modifiers play a role in the differential outcomes among these patients.

Small-conductance calcium-activated potassium (SK) currents are repolarization currents responsible for afterhyperpolarization of the neurons in the central nervous system.^[5,6]^ Recent studies have demonstrated the existence of SK currents in the atrial myocytes that are responsible for atrial repolarization.^[7–9]^ The SK current is downregulated in diabetic mouse atria and in human chronic atrial fibrillation (AF).^[10,11]^ Inhibition of SK current is proarrhythmic in healthy atria but antiarrhythmic in atrial tachypacing-induced remodeled atria due to the associated increase in action potential duration.^[12–15]^ Although SK current is either very small or undetectable in normal ventricular myocytes paced at normal rates,^[16–18]^ it is important for ventricular repolarization in normal hearts with bradycardia and/or hypokalemia, as well as in ischemic and failing ventricles.^[18–25]^ Upregulation of SK current in failing hearts is responsible for postshock action potential duration shortening and recurrent spontaneous ventricular fibrillation (VF).^[18]^ Although blocking SK current in failing rabbit hearts may act as an antiarrhythmic by suppressing electrical storm, it may also reduce repolarization reserve and induce early afterdepolarization and torsades de pointes ventricular arrhythmia.^[24]^ Therefore, the role of SK current upregulation or downregulation in ventricular arrhythmia is complex, depending on the clinical scenario.^[26,27]^ Nevertheless, these findings suggest that SK channels may play an important role in the pathogenesis of ventricular arrhythmia.

SK channels have 3 subtypes, encoded as potassium calcium-activated channel subfamily N member 1, 2, and 3 (*KCNN1*, *KCNN2*, and *KCNN3*). Among these, variants of *KCNN3* are known to be associated with AF,^[28]^ and SK channel subtype 2 (SK2) is known to be upregulated in failing human hearts and plays an important role in ventricular repolarization.^[19,20]^ Although genetic variants are known to be associated with human cardiac arrhythmogenesis,^[29,30]^ there are no reported associations between genetic variants of the *KCNN2* gene and the risk of cardiac arrhythmias. The purpose of the present study was to test the association between *KCNN2* common genetic variants and cardiac arrhythmias, including both VTa and AF.

## 2 Methods

The study protocol was in accordance with the Declaration of Helsinki and was approved by the Institutional Ethical Committee of National Taiwan University Hospital. All study subjects signed informed consent before participation.

### 2.1 Study populations

In a single tertiary referring medical center (National Taiwan University Hospital), 2 groups of patients were consecutively and prospectively enrolled. Group 1 was a defined VTa population that included the following: patients (N = 69) who received implantable cardioverter-defibrillator (ICD) implantation for secondary prevention, including survivors of cardiac arrest due to VF or hemodynamically unstable sustained ventricular tachycardia (VT) after evaluation to define the cause of the event and to exclude any completely reversible cause; and patients (N = 3) with unexplained syncope with clinically relevant, hemodynamically significant sustained VT or VF induced at electrophysiological study. Group 2 (N = 98) included patients with drug-refractory paroxysmal or persistent AF who underwent pulmonary vein isolation by radiofrequency catheter ablation. The control population (N = 144) comprised patients admitted to the Health Management Center of National Taiwan University Hospital. The latter patients did not have diabetes, hypertension, dyslipidemia, or coronary artery disease based on history, physical examination, routine laboratory testing, electrocardiogram, and chest X-ray. Due to wide range of minor allele frequencies (MAF) over different populations, only Han Chinese were enrolled.

### 2.2 Selection of SNPs and genotyping

Using the HapMap CHB databank (public data release 27 phase II + III, February 2009), 78 single nucleotide polymorphisms (SNPs) were identified in a 141-kb region containing *KCNN2*, 5 kb upstream, and 1 kb downstream using Haploview software (version 4.2).^[31]^ Twelve tag SNPs (rs6884289, rs7710366, rs2416371, rs11738819, rs10076582, rs338625, rs13181189, rs163305, rs12516818, rs1599175, rs13184658, and rs12652782) were selected, capturing 100% of haplotype variance for all SNPs on *KCNN2* with a minimum r^[2]^ value of 0.8 and MAF ≥ 5%. All SNP markers were genotyped by matrix-assisted laser desorption/ionization-time of flight mass spectrometry at the National Center for Genome Medicine, and the experimental protocol can be summarized as follows. A DNA fragment (100–300 bp) encompassing the SNP site was amplified using a polymerase chain reaction GeneAmp 9700 thermal cycler (Applied Biosystems, Foster City, CA) according to the manufacturer's instructions. After polymerase chain reaction, amplification, and neutralization of the deoxynucleotide triphosphates, primer extension was performed by adding the probe, Thermo Sequenase (Amersham Pharmacia, Piscataway, NJ), and an appropriate dideoxynucleotide triphosphate/deoxynucleotide triphosphates mixture. Extension products were differentiated by matrix-assisted laser desorption/ionization-time of flight. We then compared the reference allele frequency and MAF of our population with other populations recorded in the HapMap databank (Supplemental Table S1).

### 2.3 Statistical analysis

Baseline characteristics of the groups were compared using Student unpaired *t* test (continuous data) or chi-square test (categorical data). For each SNP, the more common allele in the controls was assigned as the reference category. A Hardy–Weinberg equilibrium (HWE) test was performed for each sequence variant of the control group before marker-trait association analysis. The association of each SNP allele with VTa or AF and control was assessed using logistic regression in models of codominant, dominant, and recessive inheritance. In the model of genotyping analysis, age and sex were adjusted by multivariant logistic regression. For further robust significant association, nominal 2-sided *P* values were corrected for multiple tests by 10,000 permutations with Haploview software.^[31]^ For haplotype construction, genotype data from the case and control groups were used to estimate intermarker linkage disequilibrium (LD) by measuring pairwise D′ and *r*^2^ and defining LD blocks. We used the confidence interval (CI) method component of the Haploview software to define an LD block with an extended spine if D′ was >0.8.^[32]^ The population attributable risk (PAR) was estimated from the control group data as follows: p × (OR − 1)/[p × (OR − 1) + 1], in which p is the prevalence of risk allele among control subjects and OR is the odds ratio of the risk allele.^[33]^ All statistical analyses were performed in IBM SPSS Statistics V20. A 2-sided *P* ≤ 0.05 was considered statistically significant.

## 3 Results

### 3.1 Baseline characteristics

In total, there were 72 patients with history of VTa and 98 patients with AF. The underlying etiologies of the VTa were dilated cardiomyopathy in 31 (43.1%), ischemic cardiomyopathy in 20 (27.8%), and non-heart failure in 21 (29.1%), including 14 (19.4%) with idiopathicVF, 3 (4.2%) with Brugada syndrome, 2 (2.8%) with arrhythmogenic right ventricular cardiomyopathy, 1 (1.4%) with hypertrophic cardiomyopathy, and 1 (1.4%) with long QT syndrome. All the other clinical characteristics are listed in Table 1. VTa is the most disastrous and common end-stage presentation of patients with various cardiovascular diseases, especially those with congestive heart failure of different etiologies. There may be a common factor that predisposes these patients to VTa. Therefore, in the present study, we enrolled a group with various underlying diseases to investigate the association of *KCNN2* SNPs with VTa.

**Table 1:**
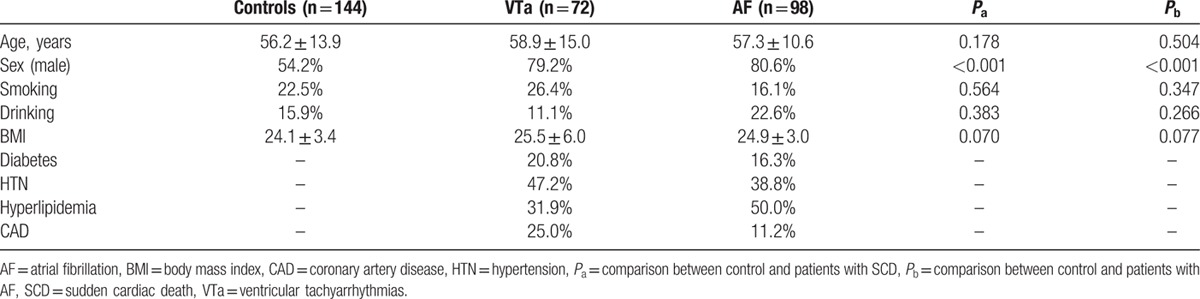
Clinical characteristics.

### 3.2 Association between *KCNN2* SNPs and VTa

**Figure 1:**
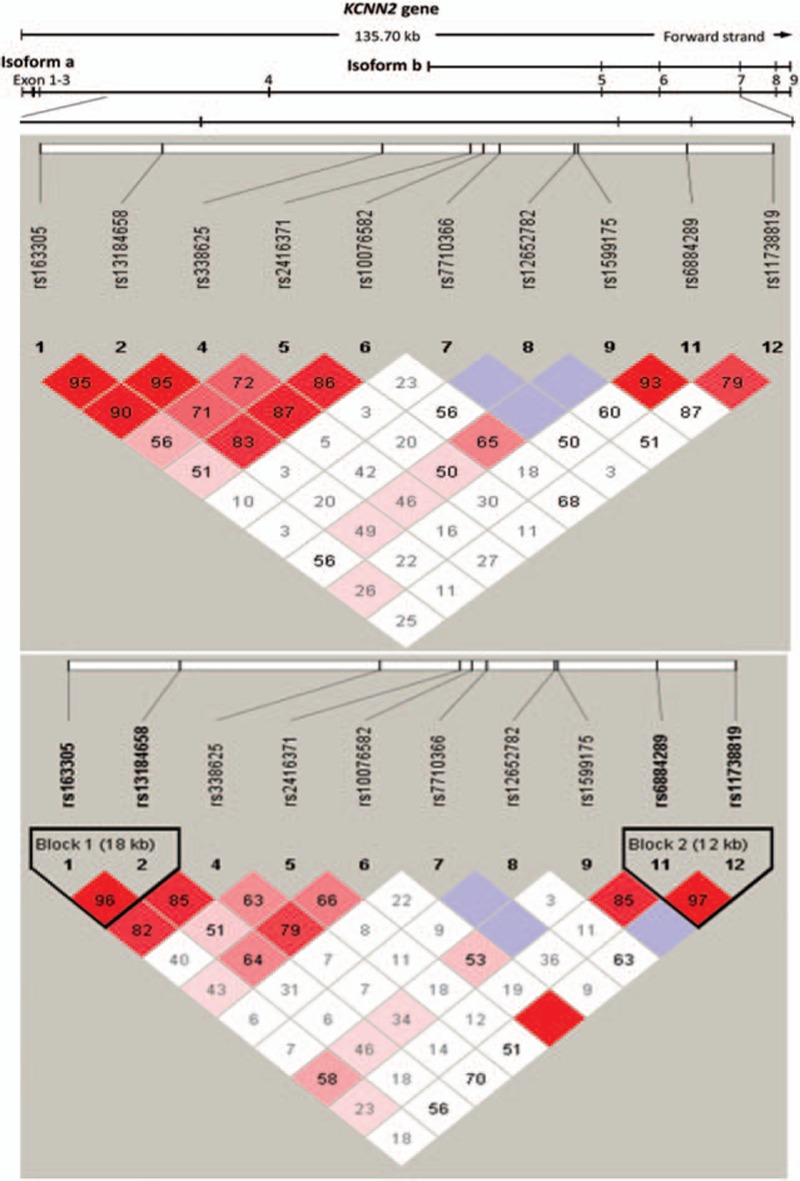
Graphical representation of single nucleotide polymorphisms in relation to the exon-intron structure (upper line) and Haploview LD graph of the *KCNN2* gene (middle and lower panels). The exon regions are shown as filled rectangles and are numbered in order. Pairwise LD coefficients D′ × 100 are shown in each cell (D′ values of 1.0 are not shown). A standard Haploview color scheme was applied to the LD color display (LOD score ≥ 2 and D′ = 1 shown in bright red; LOD score ≥ 2 and D′ < 1 shown in pink; LOD score < 2 and D′ = 1 shown in blue; LOD score < 2 and D′ < 1 shown in white). The middle panel shows the association with ventricular tachyarrhythmias; lower panel shows the association with atrial fibrillation. KCNN2 = potassium calcium-activated channel subfamily N member 2, LD = linkage disequilibrium, LOD = logarithm of odds.

There are up to 78 common SNPs in the human *KCNN2* gene. To decrease genotyping effort, we only genotyped representative tag SNPs. We selected tags that captured most of haplotype variance for all SNPs on the *KCNN2* gene. Ten tag SNPs, not including rs13181189 and rs12516818, were successfully genotyped, capturing 89% of haplotype variance. For these 10 SNPs, the success rate of genotyping was 99.7% (range: 99.1%–100%). A graphic representation of the SNPs in relation to the exon-intron structure (according to the National Center for Biotechnology Information) is shown in Fig. 1, middle panel. All the SNPs were in the introns. The genomic position, nucleic acid composition, MAF, HWE test *P* values, OR, and nominal and permuted *P-*values of the 10 genotyped SNPs are presented in Table 2. One SNP deviated from the expected count by HWE (rs12657682, *P* = 0.049).

**Table 2:**
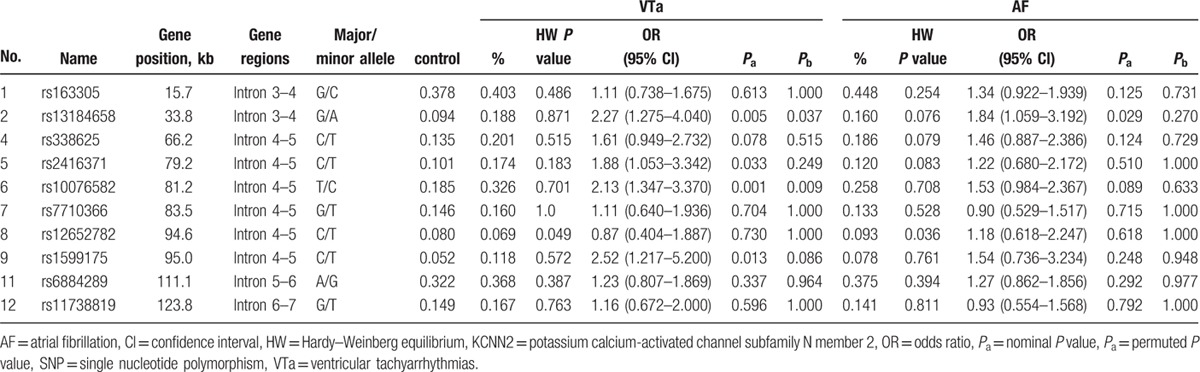
Allele association of the 10 tag SNPs in the *KCNN2* gene with the risk of sudden cardiac death.

We first compared the allele frequencies of all the SNPs of case and control patients. Interestingly, 4 variants (rs13184658, rs2416371, rs10076582, and rs1599175) showed significant association with VTa. Among these, 2 SNPs (the A variant of rs13184658 and C variant of rs10076582) remained significant after 10,000 permutations (Table 2) and Bonferroni correction. The OR of rs13184658 was 2.27 (95% CI = 1.275–4.040; permutated *P* = 0.037; *P* = 0.046 with Bonferroni correction) and that of rs10076582 was 2.13 (95% CI = 1.347–3.370, permuted *P* = 0.009; *P* = 0.011 with Bonferroni correction). The estimated PARs were 17.3% and 10.6%, respectively. There was no LD detected.

Genotypic analysis (Table 3) showed significant association of 4 SNPs (rs13184658, rs338625, rs10076582, and rs1599175) with VTa. All except rs1599175 revealed significant association in a dominant model. There was no homozygous risk allele for rs1599175. After adjustment for age and sex, all the associations remained significant. After further adjustment for other risk factors, including smoking, drinking, and body mass index, rs13184658, rs338625, and rs10076582 still showed significant association with VTa.

**Table 3:**
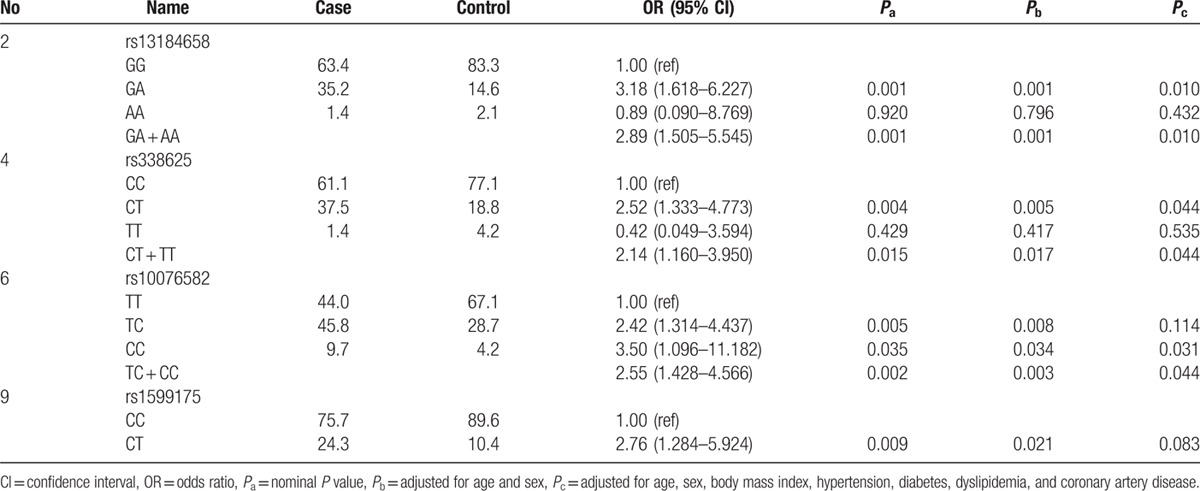
Genotype association analysis.

Further analysis was attempted to elucidate the SNP effect among subgroups relative to underlying disease by grouping them as dilated cardiomyopathy, ischemic cardiomyopathy, and non-heart failure (Table 4). However, the power of the analysis was limited due to the relatively small number of cases. Nonetheless, similar trends of positive association were still observed among all three subgroups, implicating the *KCNN2* genetic variant as a common factor predisposing patients to VTa.

**Table 4:**
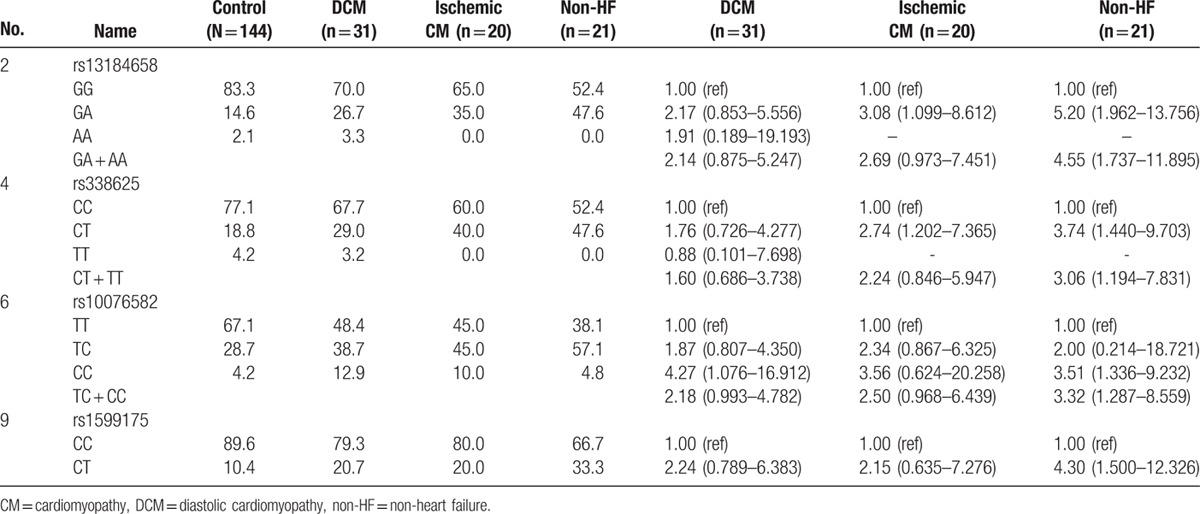
Subgroup analysis.

### 3.3 Association between SNPs and AF

Based on the standardized pairwise LD coefficient D′ and estimated *r*^2^ of the markers, 2 LD blocks were identified across the gene (Fig. 1, lower panel). One SNP (A allele of rs13184658) showed significant association with AF (OR 1.84, 95% CI = 1.059–3.192, *P* = 0.029). This SNP was located in LD block 1 in intron 3. The haplotypes within this LD block were significantly associated with AF. Among these haplotypes, the haplotype C-A was associated with an increased risk of AF (*P* = 0.02). However, both associations became insignificant after 10,000 permutations and Bonferroni correction. In a genotyping test, this variant, rs13184658, showed significant association in a dominant model (OR 1.91, 95% CI = 1.025–3.570, *P* = 0.042). After adjustment for age and sex, the associations remained significant (OR 3.20, 95% CI = 1.020–3.809, *P* = 0.044).

## 4 Discussion

We found a significant association between common *KCNN2* variants and sustained VTa in a group of consecutively enrolled patients undergoing ICD implantation for secondary prevention of SCD. Two SNPs (rs13184658 and rs10076582) showed significant association with VTa in a dominant model, carrying 2.55- to 2.89-fold increased risk. The association was robust, as it remained significant after adjustment for multiple potential risk factors. These results suggested that SK2 current may play a role in the mechanism of human VTa.

### 4.1 Previous genetic association studies with the intermediate phenotype of SCD

Earlier genome-wide association studies (GWAS) and candidate gene analyses identified many rare or common variants that are associated with intermediate phenotypes predictive of SCD risk, including quantitative ECG traits, risk of coronary artery disease, and autonomic function.^[34–37]^ However, the effects of variants associated with intermediate phenotypes were not consistent in later studies,^[38]^ suggesting heterogeneity of underlying genetic causes for those phenotypes.^[39]^ Although conducting a GWAS study to identify SCD genes directly might be a more comprehensive and attractive approach, not all risk variants can be detected by this approach due to its limited power, particularly in the regions that are not well covered by this technique. Focusing on specific candidate genes based on accumulated knowledge from in vivo and in vitro studies and directly using the targeted phenotype, as we have done in this study, is an efficient method of identifying potentially important risk allele(s).

### 4.2 Second hit theory: *KCNN2* variants predispose patients to a greater risk of SCD

Previous studies also showed that while certain rare or common variants (*AT1R*, *ADRB2*, *SCN5A*, *KCNH2*, and *NOS1AP*) might not be directly associated with significant clinical arrhythmia syndromes, they affected carriers and predisposed them to malignant arrhythmia when exposed to a 2nd hit, like heart failure or myocardial ischemia.^[40–43]^ Similar scenarios could also be seen in cases of acquired long QT syndrome and ischemic cardiomyopathy, in which rare variants (*KCNH2*, *KCNE1*, *KCNE2*, *KCNQ2*, *SCN5A*, and *RyR2* in drug-induced long QT syndrome and *CASQ2*, *RAB3GAP1*, *ZNF365*, *CXADR*, *GPC5*, *GPD1L*, *NOS1AP*, and *SCN5A* in ischemic cardiomyopathy) were associated with higher risk of malignant arrhythmia and SCD.^[44–49]^ In Brugada syndrome, the variants at SCN5A–SCN10A have a strong impact on susceptibility to SCD.^[50]^ Population-based studies have also found associations between SCD and variants in *AGTR1*, *AGTR2*, *NOS1AP*, *SCN5A*, and *ADRB2*.^[51–54]^ In the present study, although the underlying disease etiologies were diverse, a shared trait linked patients to VTa. The association of *KCNN2* variants with VTa in patients with a heterogeneous background implied a role of *KCNN2* in susceptibility to SCD when exposed to a 2nd hit (an environmental or nongenetic factor) regardless of the underlying cardiac pathology. This was also supported by the finding of our subgroup analysis that all 3 groups have similar trends of positive association.

### 4.3 Trend of association of *KCNN2* genetic polymorphisms with AF

We also found a weak association between *KCNN2* (rs13184658) and AF. An earlier GWAS study of lone AF patients showed an association with variants of *KCNN3*, another subtype of the SK family, but not with *KCNN2*.^[28,55–57]^ However, downregulation of both SK2 and SK3 currents was observed in human chronic AF,^[8]^ and ablation of SK2 channels resulted in a delay in cardiac repolarization and atrial arrhythmias.^[7]^ Overexpression of the SK3 channels in transgenic mice led to bradyarrhythmias and heart block but not to ventricular arrhythmias.^[58]^ Our finding of an association between AF and *KCNN2* further confirms that SK currents are important in the generation or maintenance of AF.

### 4.4 Potential mechanisms of the association between *KCNN2* variants and SCD

The pathophysiological roles of the associated *KCNN2* polymorphisms remain unknown. All the polymorphisms are in intron regions and are not directly transcribed to the structure of the SK2 protein. Recent studies have shown that noncoding microRNAs (miRs), which are mainly produced in the intergenic or intron regions, play an important regulating role in gene expression, and may possibly be involved in the pathophysiology of numerous cardiovascular diseases. A single miR can regulate multiple genes, and a single gene can be regulated by multiple miRs.^[59–61]^ It is possible that some of the polymorphisms in the LD block that is associated with our tag SNPs (rs13184658 and rs10076582) are true disease-associated SNPs and are involved in the mechanism of SCD at the epigenetic level mediated by miRs, affecting either *KCNN2* or other related genes’ activities. Because SK currents may serve as rescue currents that maintain repolarization reserve in disease conditions,^[26]^ alteration of SK current activity may contribute to cardiac arrhythmogenesis. However, further studies are needed to confirm or dismiss this possibility.

### 4.5 Limitations

There are several limitations to this study. First, we used the tag SNP association approach to search for common variants associated with common phenotypes among sporadic cases. This approach depends on LD to identify associated SNPs. Therefore, the true responsible variants may not be identified. Direct sequencing for all exons and introns to identify possible rare variants might be another approach, but this would dramatically increase the study cost without increasing the statistical power significantly. Family aggregation analysis would be another approach. However, we did not pursue detailed family histories or acquire blood samples from other family members. Most of the patients were sporadic cases, and we made sure that only 1 patient in each family was indexed in this study. Without knowing the exact responsible variant, further in-depth functional study was not possible. Second, the number of cases in this study is relatively small. Expanding the case numbers for this study or repeating the study results in another independent group of patients is another attractive option. Because it is not easy to clarify the association between SK2 and ventricular arrhythmias, we decided to recruit only patients with the most extreme phenotypes (lethal ventricular arrhythmias) who received ICD implantation for secondary prevention. Expanding the patient group to hemodynamically stable VTs or other intermediate phenotypes might increase the probability of false negative results. Third, the frequency of the variants in a population can affect the PAR and may vary significantly among different populations (Supplemental Table S1). It appears that most *KCNN2* gene variants have much lower frequency in Han Chinese than in Caucasians. It is possible that the *KCNN2* variants reported in this cohort are not associated with arrhythmias in other populations. Validation of our findings in other populations, particularly among the Caucasians, is warranted. Last, while we attempted to capture 100% of the haplotype variance using 12 tagging SNPs, we failed to genotype 2 of these. Therefore, we only analyzed 10 SNPs, capturing 89% of the haplotype variance. Among the 10 variants, 1 (rs12652782) deviated from HWE. This may be due to the low frequency of the minor allele (as shown in Table 2 and the Supplemental Table S1), a small sampling size, or both.

## 5 Conclusion

Variants of the *KCNN2* gene are associated with the occurrence of lethal ventricular arrhythmias in patients with underlying heart diseases. The clinical impact of these results remains unclear and deserves further study.

## Supplementary Material

Supplemental Digital Content
