# Supplementary material for: KCNN2 polymorphisms and cardiac tachyarrhythmias
Source: Medicine (Baltimore). 2016 Jul 22;95(29):e4312. doi: 10.1097/MD.0000000000004312 (PMC5265796; doi:10.1097/MD.0000000000004312)
Supplement: Supplemental Digital Content [file medi-95-e4312-s001.doc]

Supplemental Table S1. Comparison of the frequencies of *KCNN2* gene variants in our cohort with other populations.

| No. | Name | Major/minor allele | Our cohort | MAF of CHB | MAF of CEU | MAF of JPT | MAF of YRI |
| --- | --- | --- | --- | --- | --- | --- | --- |
| 1 | rs163305 | G/C | 0.375 | 0.601 | 0.177 | 0.616 | 0.119 |
| 2 | rs13184658 | G/A | 0.094 | 0.156 | 0.347 | 0.089 | 0.086 |
| 3 | rs13181189 | G/A |  | 0.167 | 0.271 | 0.081 | 0.045 |
| 4 | rs338625 | C/T | 0.135 | 0.185 | 0.389 | 0.134 | 0.814 |
| 5 | rs2416371 | C/T | 0.101 | 0.125 | 0.168 | 0.076 | 0.066 |
| 6 | rs10076582 | T/C | 0.184 | 0.220 | 0.416 | 0.174 | 0.823 |
| 7 | rs7710366 | G/T | 0.146 | 0.167 | 0.544 | 0.186 | 0.872 |
| 8 | rs12652782 | C/T | 0.080 | 0.120 | 0 | 0.128 | 0 |
| 9 | rs1599175 | C/T | 0.052 | 0.079 | 0.055 | 0.054 | 0 |
| 10 | rs12516818 | A/G |  | 0.399 | 0.783 | 0.366 | 0.704 |
| 11 | rs6884289 | A/G | 0.319 | 0.342 | 0.464 | 0.291 | n/a |
| 12 | rs11738819 | G/T | 0.149 | 0.161 | 0.310 | 0.163 | 0.097 |

CEU, Utah residents with ancestry from northern and western Europe ancestry from the CEPH collection; CHB, Han Chinese in Beijing, China; JPT, Japanese in Tokyo, Japan; YRI, Yoruba in Ibadan, Nigeria. Variants #3 (rs13181189) and #10 (rs12516818) were failed to be genotyped.
